# The role of oxidative stress in determining prognosis in children with FMF and the relationship between markers of oxidative stress and gene mutation

**DOI:** 10.1186/1546-0096-13-S1-P107

**Published:** 2015-09-28

**Authors:** O Eryavuz, R Dusunsel, I Dursun, K Köse, H Poyrazoglu, Z Gunduz, S Yel

**Affiliations:** 1 Erciyes University Faculty of Medicine, Pediatric Rheumatology, Kayseri, Turkey; 2 Erciyes University, Faculty of Medicine, Department of Biochemistry, Kayseri, Turkey

## Background

Uncontrollable neutrophil activation and its migration to serous tissues that result in oxidative stress responsible for the tissue damage due to pyrin mutation in FMF patients. Reactive oxygen radicals are released by active neutrophils and the basic cell compounds such as nucleic acids, proteins and lipids during the inflammation. The aim of our study was to measure plasma advanced oxidation protein product (AOPP) and serum amyloid A (SAA) in children with FMF and to investigate the relationship with clinical findings and gene mutations as well.

## Material and methods

One-hundred nineteen patients with FMF patients aged between 7 and 15 years and 29 age-and sex-matched healthy controls were enrolled in the study. Patients were classified based on response to colchicine. Briefly, group 1; 29 children taking colchicine without any attack, group 2; 30 children with frequent attack despite taking colchicine, group3; 30 children taking colchicine with unchanged frequency of attack, group 4; 30 children newly diagnosed as FMF and not taking colchicine, group 5; control. Acute phase reactants including ESR, CRP and SAA and were measured at the beginning of study and plasma AOPP levels were as well. The patients were also evaluated according to the presence or absence of attacks and the correlation of clinical and genetic features with oxidative stress markers were investigated.

## Results

AOPP levels of group 2, 3 and 4 were significantly higher than group 1 and control. There was statically significant different between patients and control in terms of the SAA levels. 80 % of the patients in group 3 and 43.3 % of the patients in group 4 had SAA level >6.4 mg/L. The patients having FMF attacks at the time of study had significantly higher SAA and AOPP levels than patients being attack free and control. Regardless of FMF attack, patients with FMF had higher level of AOPP and SAA than control. Patients bearing the M694V/M694V genotype had high level of SAA. There was no significant difference between patients with and without M694V/M694V genotype in terms of AOPP level.

## Conclusion

The present study demonstrates that children with FMF have high level of AOPP that reflects the oxidative damage of proteins and level of SAA that thought to be a marker of both a chronic inflammation and the activation in FMF.

